# SGLT2 Inhibitors in Clinical Practice: Cardiorenal Benefits and Risk of Fungal Infections—A Nephrologist’s Perspective

**DOI:** 10.3390/jcm15093395

**Published:** 2026-04-29

**Authors:** Wiktoria Andryszkiewicz, Zuzanna Cichowska, Maria Kręcicka, Sławomir Zmonarski

**Affiliations:** 1 Faculty of Medicine, Wroclaw Medical University, 50-367 Wroclaw, Poland; wiktoria.andryszkiewicz@student.umw.edu.pl (W.A.); zuzanna.cichowska@student.umw.edu.pl (Z.C.); maria.krecicka@student.umw.edu.pl (M.K.); 2 Department of Nephrology, Transplantation Medicine and Internal Diseases, Wroclaw Medical University, 50-367 Wroclaw, Poland

**Keywords:** SGLT2 inhibitors, diabetic kidney disease, urinary tract infection, genital mycotic infection, candiduria

## Abstract

People affected by diabetes mellitus (DM), chronic kidney disease (CKD), or heart failure are often prescribed sodium–glucose cotransporter-2 inhibitors (SGLT2is) as a method of treatment. These drugs favorably affect glucose metabolism, as patients taking them have lower serum glucose levels. Another promising positive impact is protection against microvascular damage, cardiovascular disease, and chronic kidney disease. Nevertheless, fungal infections, urinary tract infections, and ketoacidosis are the adverse effects that might happen during the SGLT2i treatment. Fungal infections are more common among patients treated with these medications, including vulvovaginal Candidiasis in women and balanitis or balanoposthitis in men. Given the difficulty of treating fungal infections in patients with co-occurring diseases, we recommend that ongoing supervision of patients treated with SGLT2i include prevention and early detection of fungal infections.

## 1. Introduction

The range of clinical indications for sodium–glucose cotransporter type 2 inhibitors (SGLT2is) is steadily expanding. SGLT2is were originally used to lower glucose levels in patients with type 2 DM (DM2). Further trial evidence and clinical guideline updates have demonstrated their cardiovascular and renal benefits. This evidence led to the widespread use of SGLT2i [1,2,3,4,5]. Many indications for SGLT2i use encompass conditions that independently predispose to urogenital fungal infections. In general, people who are especially prone to fungal urinary tract infections (UTIs) include women with diabetes mellitus (DM), patients with urinary tract obstruction such as nephrolithiasis, and those with urinary tract devices such as Foley catheters and stents, as well as renal transplant recipients [6]. Symptomatic Candida infection, the most common, is more likely in female patients, diabetic patients, pregnant females, and individuals using hormonal contraceptives, antibiotics, or immunosuppressants [7]. *Candida* spp. can cause funguria, renal *Candida*, and *Candida* pyelonephritis [6]. Although genital fungal infections are seldom dangerous, they may significantly affect quality of life, especially in patients with comorbid conditions and functional impairments [8]. DM2 itself leads to various impairments, such as autonomic neuropathy, including diabetic bladder dysfunction and incomplete bladder emptying, which can lead to recurrent urinary tract infections, especially if glucose levels are not properly controlled [9,10,11]. Persistent glycosuria in DM patients makes them more susceptible to genital mycotic infections [12].

Physicians must understand the mechanism of action of SGLT2is and apply this knowledge to optimize patient outcomes while balancing the risk of adverse effects. SGLT2is in non-diabetic patients without hypoglycemia may lead to various adverse effects, such as hypotension secondary to excessive diuresis and natriuresis. [13]. SGLT2i use results in an average urinary glucose excretion of 40–80 g/d in patients without DM. In patients with present DM2, these values are usually higher [14]. The side effects, among others, include acute kidney injury (AKI), increased urination, urinary tract infection, and genital fungal infections. SGLT2i-related glycosuria predisposes to urogenital infections, which are more prevalent in females. The risk of vulvovaginal mycosis increases 2- to 4-fold in women, especially in those treated with dapagliflozin [12]. Glycosuria creates an environment favorable for the growth of numerous pathogens, especially *Candida* spp. Among them, *Candida albicans* can be distinguished by its ability to grow in a milieu opulent in glucose, which allows a glucose-activated protein to enhance adhesion and hinder phagocytosis by the immune system [8,15,16,17].

## 2. Materials and Methods

### 2.1. Search Strategy

This manuscript is a narrative review. A literature search was conducted on databases such as PubMed, Scopus, and Web of Science. The search included studies published in English, using keywords such as: “SGLT2 inhibitors”, “fungal infections”, “candidiasis”, “urinary tract infections”, and “chronic kidney disease”.

### 2.2. Inclusion and Exclusion Criteria

Studies were included if they addressed the use of SGLT2 inhibitors in relation to fungal and genitourinary infections, as well as cardiorenal benefits. We considered randomized controlled trials, observational studies, meta-analyses, clinical guidelines, and relevant English-language review articles. Studies not directly relevant to the scope of this review and lacking clinical significance were excluded.

### 2.3. Literature Selection

Priority was given to recent publications, particularly from the last 5–10 years, although older studies were included when considered clinically or scientifically relevant. Key studies were included where necessary to provide essential background and context. Both randomized controlled trials and observational studies, as well as review articles, were analyzed. The selection of articles was based on their relevance to the topic, methodological quality, and contribution to understanding the relationship between SGLT2 inhibitors and fungal infections.

## 3. Mycotic Infection in a Patient Treated with SGLT2is—Prevalence, Diagnostic and Therapeutic Pathway, and Prognosis

### 3.1. Prevalence of SGLT2i-Related Mycotic Infections and Clinics

In general, according to various studies, the prevalence of urogenital infections varies slightly across studies [18]. In a study from August 2020, 7.8% patients with DM2 experienced a genitourinary infection, with 98% occurring within the first few months of treatment [7]. In another study, empagliflozin had the highest number of reported fungal infections, followed by canagliflozin and dapagliflozin. Ertugliflozin showed the lowest rate [9]. Combined therapy with SGLT2is and metformin may reduce the risk of UTIs and fungal infections [9]. In some SGLT2is (dapagliflozin), the risk of UTI is dose-related in both sexes [7,13]. The flozin-related UTI may develop within the first month of therapy and continue throughout. A notably higher likelihood was found after using canagliflozin, ertugliflozin, empagliflozin, remogliflozin, dapagliflozin, and sotagliflozin, whereas no such effects were observed with the use of luseogliflozin and ipragliflozin [8,15,17].

In cases of urinary tract infections, most patients with candiduria are asymptomatic. Mycotic cystitis or pyelonephritis may cause non-specific symptoms. However, it might cause antibiotic-resistant fever. In poorly controlled DM, pneumaturia, fungus balls, and bezoars may be present, and urethral obstruction symptoms are expected. Cystoscopy and ultrasonography of the kidneys and bladder are helpful [16,19]. Among various trials involving SGLT2is, genital infections continue to be the most consistent finding. They are more common in females, in which case the manifestation is vulvovaginal infection. In males, it is balanitis or balanoposthitis [12]. According to a study from August 2018, canagliflozin was associated with a significantly higher risk of genital mycotic infections compared to sitagliptin [20]. Prostatitis and epididymo-orchitis with *Candida* etiology may present clinically similarly to bacterial infections. In epididymo-orchitis, the symptoms tend to be prolonged from 5 days to 5 months and are usually bilateral. *Candida* prostatitis can mimic prostate cancer and cause lower urinary tract symptoms; it can also coexist with prostate cancer and sexual dysfunction, but rarely presents with acute abscess [19].

### 3.2. Diagnostic Pathway

There is no standardized definition of candiduria. Separate urine Candida colony counts do not enable differentiation between contamination, colonization, and infection. The laboratory methods available are microscopic examination (^3^10 leukocytes/mm^3^) or the leukocyte esterase test. The growth of at least one culture of urine yielding ≥1 × 10^3^ colonies/mL indicates candiduria [11,19]. Differentiation between colonization and infection requires evidence of tissue reaction. Blood cultures for *Candida* are often negative [16].

**Figure 1:**
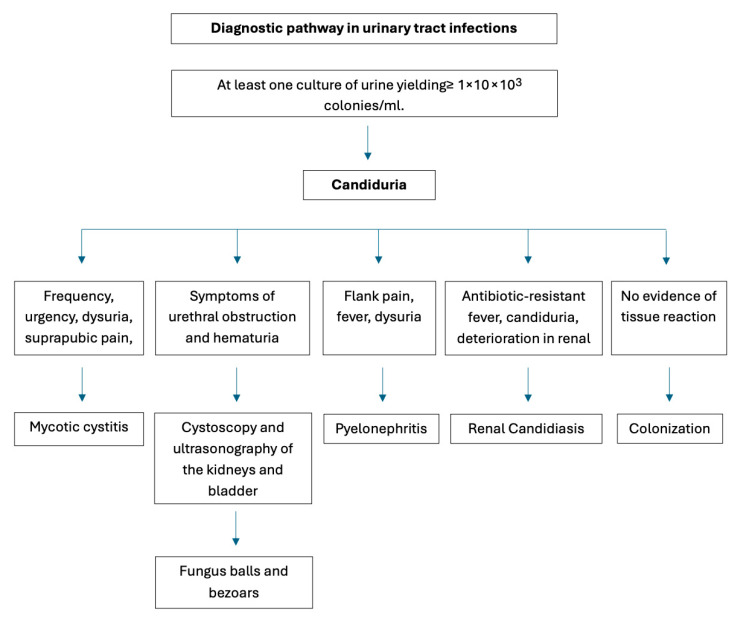
Suggested diagnostic pathway for identifying fungal urinary tract infections (authors’ original work).

**Figure 2:**
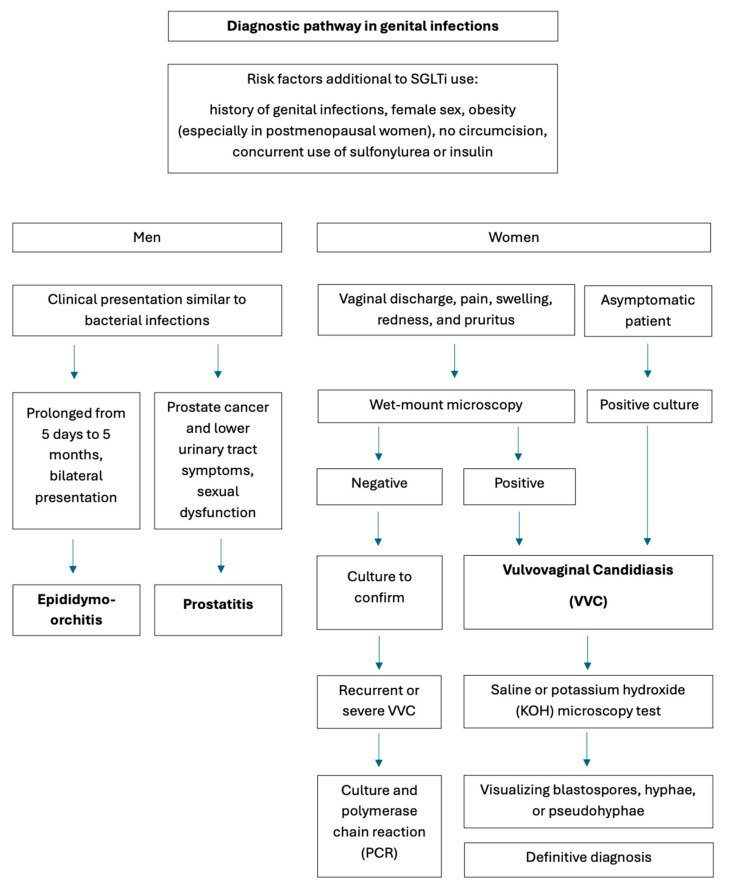
Suggested diagnostic pathway for identifying genital fungal infections (authors’ original work).

The diagnosis of vulvovaginal Candidiasis (VVC) is challenging because its symptoms are not characteristic of a mycotic etiology and may resemble those of common bacterial vaginosis or trichomoniasis. Therefore, clinical diagnosis is unreliable and should be confirmed by testing, such as wet-mount microscopy, potassium hydroxide test, vaginal pH test, culture, or polymerase chain reaction (PCR). According to guidelines, all women presenting with VVC symptoms should have wet mount microscopy followed by culture if results are negative. In severe or recurrent VVC, culture and PCR are recommended [21]. Visualization of blastospores, hyphae, or pseudohyphae on saline or potassium hydroxide (KOH) microscopy is the definitive diagnosis of VVC, or a positive culture in a symptomatic patient. Wet-mount microscopy excludes the presence of clue cells, indicating bacterial etiology or motile trichomonads. However, microscopy sensitivity is poor; as many as half of patients with culture-positive symptomatic VVC have negative microscopy results, especially in cases of *Candida glabrata* infection [22]. Risk factors other than SGLT2is include a history of genital infections, obesity (especially in postmenopausal women), uncircumcised men, concurrent use of sulfonylurea or insulin, and make patients more susceptible to fungal GUI. For such patients, screening for infections should be part of every appointment [12]. In Figure 1 and Figure 2, an example of a diagnostic pathway for mycotic infections is shown [15,20].

### 3.3. Management of SGLT2i-Related Urogenital Mycotic Infections and Prognosis

The cornerstone of effective therapy is tailoring it to each patient’s circumstances and achieving better glycemic control [22]. Patients with volume depletion should be closely monitored, and the drug dose adjusted, especially in those with high-risk factors. In case of diabetic ketoacidosis and hypoglycemia, patients require education about the symptoms, as it enables early recognition. These patients need blood monitoring and insulin, if required [23].

Persistent candiduria in an asymptomatic patient is a sign to evaluate possible predisposing factors, such as a bladder catheter, the removal of which is usually a sufficient treatment. Antifungal treatments are recommended if the patient is symptomatic or neutropenic, early after renal transplantation, in cases of very low birth-weight infants (<1500 g), and if the patient is undergoing urologic procedures. Later, after renal transplants, treatment of asymptomatic candiduria is not recommended. In treating symptomatic patients, fluconazole is the drug of choice, especially given its high renal excretion (up to 80% of the dose). However, it poses the risk of interactions, liver toxicity, QT interval prolongation, *Candida glabrata* resistance, and *Candida krusei*. Drug resistance poses a significant clinical challenge; the choice of antifungal agent should be guided by susceptibility testing and consultation with an infectious disease specialist. A combination of antifungal drugs and rotating therapies may be implemented to manage resistant infections. In such cases, amphotericin B or flucytosine is often added [15,16,19].

An essential factor to consider is the level at which the drug is excreted into urine. Unlike fluconazole, other antifungals do not reach high concentrations in urine and are not recommended for treating cystitis. However, posaconazole and voriconazole concentrate well in kidney tissue and can be effective treatment choices for *Candida* renal parenchymal infections [19]. Patients with fungal pyelonephritis need to be hospitalized. In cases involving fungus balls, surgical treatment is necessary. These severe conditions require the discontinuation of SGLT2is and their avoidance in the future [24]. In terms of dosages, the following apply: for cystitis, fluconazole 200 mg orally once daily; for pyelonephritis, 200–400 mg orally once daily. If the pyelonephritis is resistant, flucytosine 25 mg/kg orally 4 times a day is added. The dosage needs to be adjusted based on the current creatinine clearance [16]. When candiduria is accompanied by candidemia, the patient’s hemodynamic status should be evaluated. If the patient is septic or has recently received azole antifungals, echinocandins are the drugs of choice. It can be replaced with fluconazole on the fifth day of treatment, provided the pathogen is susceptible, and the patient is stable.

The incidence of genital infections is significantly reduced by reinforcing perineal hygiene, and prompt treatment is associated with better outcomes. Therefore, a high index of clinical suspicion is warranted during the first 3–6 months of SGLT2i therapy. For treating *Candida* balanitis, no studies on patients receiving SGLT2is have shown a superiority of any specific topical or oral regimen [15]. Achieving reasonable glucose control is the foundation for successful clinical management of *Candida* balanitis [22]. Topical antifungal agents (clotrimazole 1% cream, miconazole 1% cream, topical imidazoles) or an oral dose of fluconazole are first-line treatments and should be discussed with the patient. Testing for *Candida* and empiric treatment should be offered to female sexual partners of the patient with balanitis [15,25]. *Candida* prostatitis requires both a surgical and systemic antifungal approach. Epididymo-orchitis is usually treated with surgical intervention and fluconazole [26]. In *Candida* vulvovaginitis, if uncomplicated, it can be treated with oral and topical azoles or triterpenoids [15]. Vaginal Candidiasis has a higher incidence in pregnant women than in non-pregnant women. The number of antifungal agents considered safe during pregnancy is limited. Amphotericin B can be used to treat invasive fungal infections. Fluconazole may be very harmful to the fetus and cause spontaneous abortions. Voriconazole is also teratogenic [19].

According to the UK Kidney Association Clinical Practice Guideline, SGLT2is should be temporarily discontinued when treating acute pyelonephritis or urosepsis and stopped for patients being counseled on the symptoms of Fournier’s gangrene. It also states that SGLT2i therapy can be continued during treatment of mycotic genital infections, and patients with a history of recurrent mycotic genital infections could be given prophylactic antifungal treatment [27].

### 3.4. Prognosis

**Figure 3:**
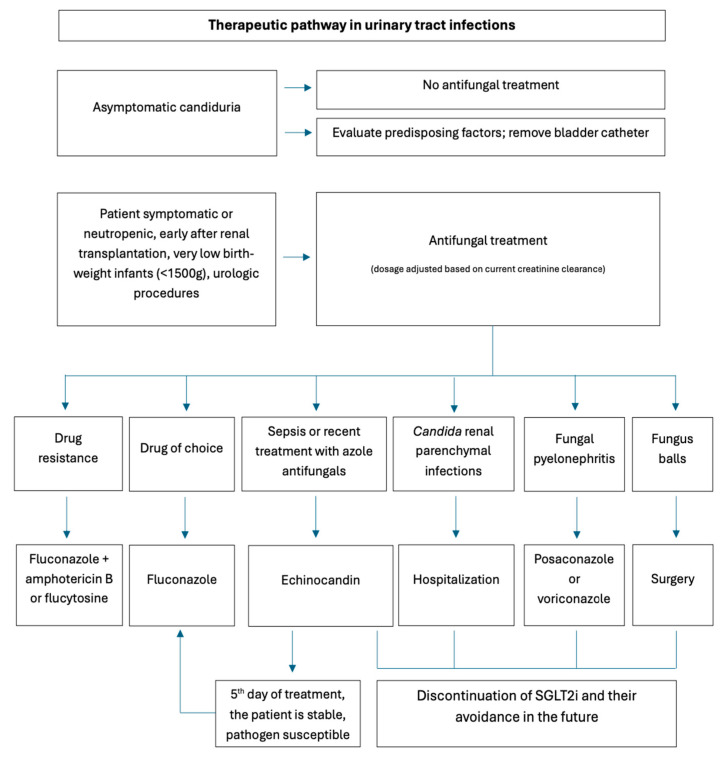
Clinical recommendations for managing urinary tract infections in patients treated with SGLT2is (authors’ original work).

**Figure 4:**
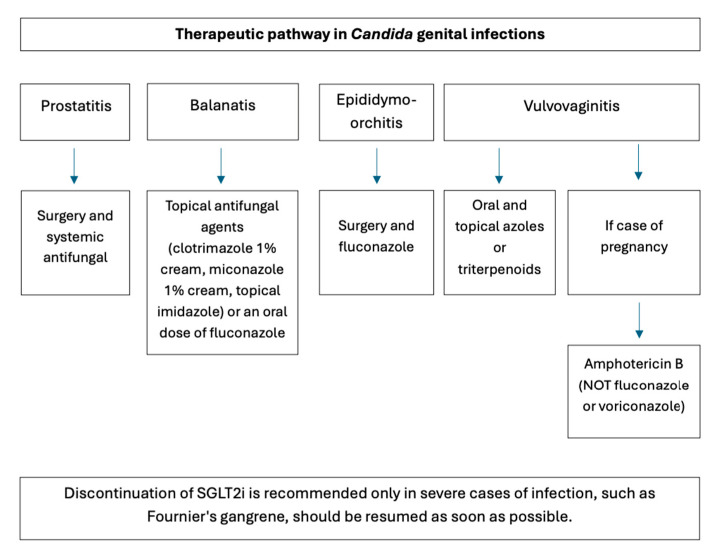
Clinical recommendations for managing *Candida* genital mycotic infections in patients treated with SGLT2is (authors’ original work).

Discontinuation of SGLT2is is recommended only in severe cases of infection. It should be resumed as soon as possible, as its benefits can be lost within a few weeks, and it can pose cardiorenal risk [15]. Patients receiving SGLT2is should be offered care plans including strategies to reduce the risk of genital Candidiasis. Effective management of genital mycotic infections can help prevent unnecessary discontinuation of SGLT2i therapy (Figure 3 and Figure 4) [7]. To conclude, the overall prognosis is generally good; infections are usually mild to moderate, respond to standard antifungal therapy, and rarely require discontinuation of the SGLT2i [18].

## 4. Conclusions and Summary

SGLT2is are drugs used to treat an expanding spectrum of conditions, such as DM2, CKD, or heart failure. Despite the numerous benefits of treating these disorders, our review shows that susceptibility to fungal infections is increased in patients using SGLT2is. Moreover, fungal infections are more frequently reported in female patients than in male patients. In general, the overall prognosis is good; the infections are usually mild to moderate, respond to standard antifungal therapy, and rarely require discontinuation of the SGLT2is. The overall benefit-risk balance remains clearly favorable. But, given the challenges of treating fungal infections in patients with co-occurring diseases, we recommend that ongoing patient supervision for SGLT2i-treated patients include prevention and early detection. Nevertheless, it is very important to remember that aside from an increased chance of developing mycotic infections, other serious complications may occur, endangering the patient’s life and resulting in severe health problems. Because of that, SGLT2i administrations must be strictly controlled. Patients are advised to receive specific strategies and proper recommendations about genital hygiene, diet, lifestyle, drug administration, and risk factors to avoid further complications. Each decision to use drugs from the SGLT2i group ought to be made with caution and taken into consideration, considering whether the risk and potential complications do not exceed the benefits of their use.

## Data Availability

The original contributions presented in this study are included in the article. Further inquiries can be directed to the corresponding author.
